# Exploring dynamics of protein structure determination and homology-based prediction to estimate the number of superfamilies and folds

**DOI:** 10.1186/1472-6807-6-6

**Published:** 2006-03-20

**Authors:** Ruslan I Sadreyev, Nick V Grishin

**Affiliations:** 1 Howard Hughes Medical Institute/Department of Biochemistry, University of Texas Southwestern Medical Center, 5323 Harry Hines Blvd, Dallas, TX 75390-8816, USA

## Abstract

**Background:**

As tertiary structure is currently available only for a fraction of known protein families, it is important to assess what parts of sequence space have been structurally characterized. We consider protein domains whose structure can be predicted by sequence similarity to proteins with solved structure and address the following questions. Do these domains represent an unbiased random sample of all sequence families? Do targets solved by structural genomic initiatives (SGI) provide such a sample? What are approximate total numbers of structure-based superfamilies and folds among soluble globular domains?

**Results:**

To make these assessments, we combine two approaches: (i) sequence analysis and homology-based structure prediction for proteins from complete genomes; and (ii) monitoring dynamics of the assigned structure set in time, with the accumulation of experimentally solved structures. In the Clusters of Orthologous Groups (COG) database, we map the growing population of structurally characterized domain  families onto the network of sequence-based connections between domains. This mapping reveals a systematic bias suggesting that target families for structure determination tend to be located in highly populated areas of sequence space. In contrast, the subset of domains whose structure is initially inferred by SGI is similar to a random sample from the whole population. To accommodate for the observed bias, we propose a new non-parametric approach to the estimation of the total numbers of structural superfamilies and folds, which does not rely on a specific model of the sampling process. Based on dynamics of robust distribution-based parameters in the growing set of structure predictions, we estimate the total numbers of superfamilies and folds among soluble globular proteins in the COG database.

**Conclusion:**

The set of currently solved protein structures allows for structure prediction in approximately a third of sequence-based domain families. The choice of targets for structure determination is biased towards domains with many sequence-based homologs. The growing SGI output in the future should further contribute to the reduction of this bias. The total number of structural superfamilies and folds in the COG database are estimated as ~4000 and ~1700. These numbers are respectively four and three times higher than the numbers of superfamilies and folds that can currently be assigned to COG proteins.

## Background

The number of currently solved protein structures [1] is about two orders of magnitude lower than the number of known amino acid sequences [2,3]. Despite intensifying efforts in protein structure determination, particularly structural genomic initiatives (SGI) [4,5], this large gap will probably remain for a considerable period of time. In protein evolution, structure tends to be much more conserved than sequence, and sequence-based inference of homology usually indicates structural similarity between proteins [6-13], exceptions to this rule being very rare [14]. There are, however, numerous cases of remote evolutionary relation undetectable by sequence and clear from the comparison of structures. Furthermore, non-homologous proteins can acquire similar structure topology (fold) as a result of structural convergence. Given all these scenarios, complete genomic sequence information alone is insufficient for a detailed classification of the protein world, which can be achieved by a comprehensive experimental determination of structures. However, using the currently known fraction of protein structures, it is possible to analyze the relations between sequence- and structure-based groupings, and to extrapolate these relations to the whole set of genomic sequences. This extrapolation may allow estimating important general features of the whole protein world, such as the total number of superfamilies of remote structure-based homologs, the total number of folds, the distribution of sequence-based families among superfamilies and folds, etc. Knowledge of these features (i) provides better understanding of evolution and current diversity within the protein universe, and (ii) sets benchmarks for structural genomic efforts to sample the whole variety of protein structures.

Several groups have analyzed these features, producing widely varying estimates of 1000 to 50000 total sequence-based families comprising 400 to 10000 folds [15-27]. Recently taken approaches [19,20,25,26] were parametric: they assumed a certain random model for the distribution of sequence-based protein families between different folds and estimated the parameters of this distribution by fitting to current structural data. Using these parameters and the estimated total number of sequence families, the total number of protein folds was derived. Although the suggested distributions often produce a very good fit for the classification of known structures, the parametric approach has several drawbacks: such estimates depend on the assumed random model, the parameters of the chosen distribution are frequently sensitive to aberrations in the used data, and can potentially change in time, with more structural data accumulated.

A related problem that has not been fully addressed in the past is the systematic bias in the selection of targets for structure determination. An assumption of previous parametric approaches is that the current set of structurally characterized families represents an unbiased random sample of all families. This assumption may potentially be wrong, for example due to the greater attention of the structural biology community to more prominent families of wider biological importance. Is the set of all currently known structures biased? Is there a bias in target sampling by SGI? How has SGI affected the bias in the overall population? This is one set of questions that we approach in this article. We find that, compared to the whole family set, the population of currently solved families has a systematic bias, which decreases with time as more structures are solved. The population of families that have been initially solved by SGI does not have an apparent bias, but this population so far comprises a minor fraction of all solved families.

Another set of questions concerns general composition of the whole set of protein structures. Here, we combine the inference of relations between sequence domains from 66 complete genomes represented in the COG database [28,29] with homology-based structure prediction, and analyze the dynamics of structure prediction for sequence families over the last 10 years. In this analysis, we assume neither a specific form of random model nor unbiased representation of the whole protein set by the families with known structures. However, we assume that the current set of these families includes a considerable statistical sample even for under-represented family categories. We also assume that the sampling bias, if it exists, changes gradually and relatively slowly in time, so that it is possible to make predictions of sampling for the future. These assumptions are supported by currently observed data. Based on our analysis, we estimate the total number of structure-based superfamilies and folds in the COG database as ~4000 and ~1700, which is respectively four and three times higher than is currently assigned to the COG database.

## Results

**Figure 1:**
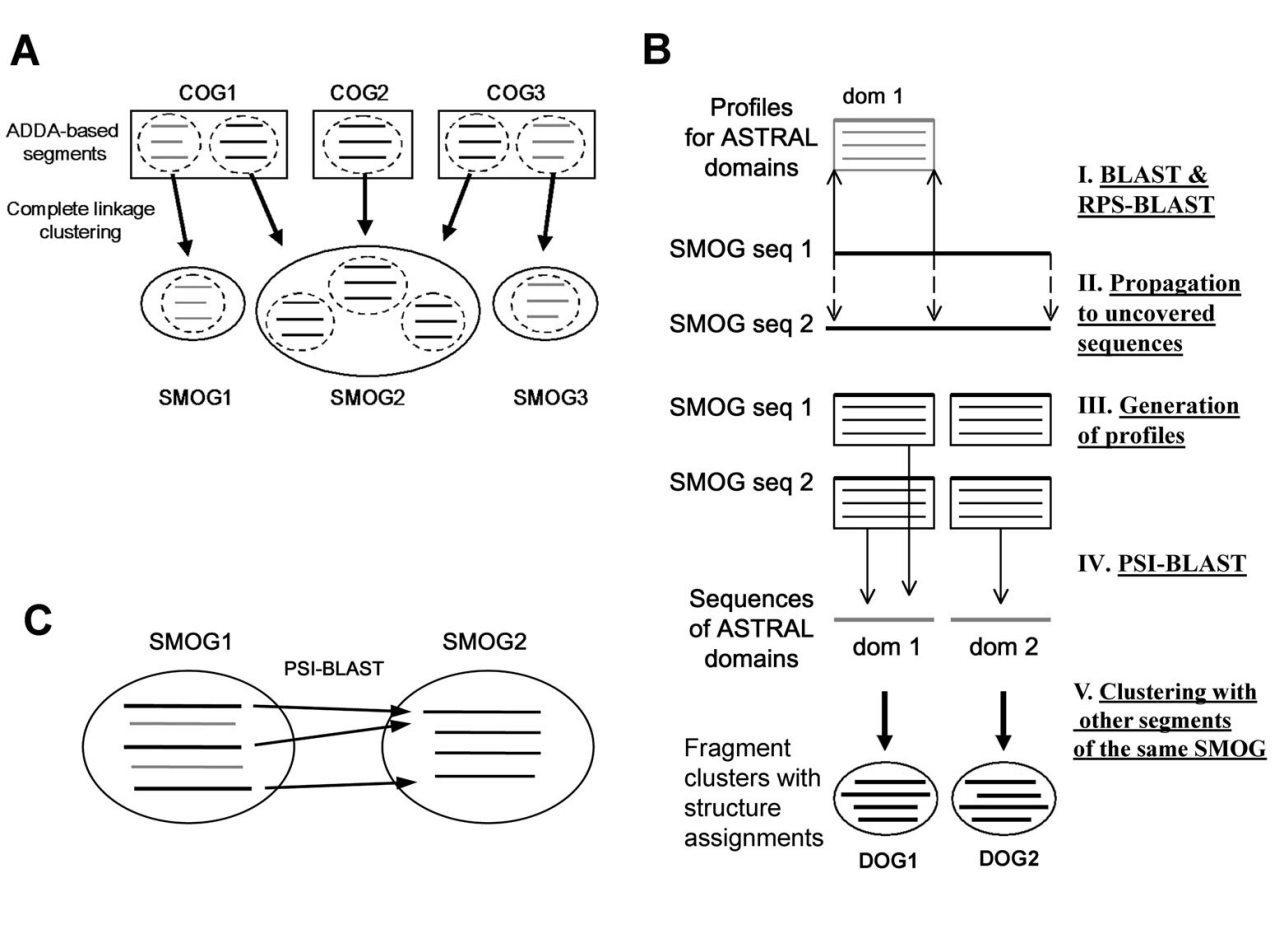
**Clustering and structure prediction for sequence domains**. A. Formation of SMOGs. Individual proteins in each COG are split in sequence-based domains using ADDA database. The resulting sequence segments are grouped by sequence similarity within each COG; then these groups from different COGs are further clustered by complete linkage. The produced clusters comprise sequence modules from orthologous groups of proteins (SMOGs), which are used as elementary units for structure assignment and sequence-based clustering (see Methods for details). B. Structure prediction in SMOG sequences. Main steps of the procedure are labeled on the right. First, individual SMOG segments are compared to sequences and profiles for SCOP representatives from ASTRAL. Using alignments between members of the same SMOG, structure assignments at the SCOP superfamily level are propagated to the regions in the SMOG segments that are not directly linked to SCOP domains. These initial assignments are used to split SMOG segments into smaller fragments, generate PSI-BLAST profiles for these fragments, and perform PSI-BLAST searches against the database of SCOP domain sequences. These searches improve the precision of the initial assignments and produce additional assignments. In a given SMOG, regions with the same superfamily assignment are clustered with other regions of this SMOG, based on PSI-BLAST alignments of SMOG sequences to each other. These clusters are referred to as DOGs (see Methods for details). C. Formation of links between SMOGs. SMOGs 1 and 2 are linked based on the fraction *W *of queries from SMOG 1 that provide detection of sequences from SMOG 2 with E-value cutoff *E*. In the shown example, *W *= 3/5 = 0.6. If all individual hits have E-value lower than *E*, the link will be formed for *W *cutoffs lower than 0.6 (e.g. *W *= 0.5), but not for higher cutoffs (e.g. *W *= 1.0).

To identify independently recurring segments in COG sequences, we use the ADDA database [30,31]. Similar ADDA-based segments within each COG are grouped together, followed by complete linkage clustering of segments from different COGs (see Methods and Fig. 1A). This clustering produces 13511 SMOGs (Sequence Modules from Orthologous Groups of proteins) in total 4873 COGs.

### Statistics of structure prediction

To make structure predictions for SMOG sequences, we use all individual SMOG segments as queries for BLAST, RPS-BLAST and PSI-BLAST [32,33] searches against (i) the ASTRAL [34,35] representatives of structural domains and (ii) other SMOG segments. These searches allow us to (i) map SCOP domains on the regions in SMOG fragments and (ii) map different SMOG fragments on each other and propagate structure predictions between highly similar regions within the same SMOG (see Methods for details). In each SMOG, we consider sequence regions that are assigned to the same SCOP superfamily and cluster these regions by sequence similarity, forming "Domains from Orthologous Groups of proteins" (DOGs, see Methods).

Based on the similarity to domains in SCOP 1.67, structure was assigned to sequence segments in 3922 SMOGs (29% of all SMOGs) that belong to 2625 COGs (54% of all COGs). Among individual sequence representatives with less than 50% identity in each COG, full-length or partial assignments were made for 66% sequences. The general statistics of structure assignments for genomes of various taxa is shown in Table 1. Similar regions assigned to same SCOP superfamilies were clustered in 7100 DOGs. In the majority of SMOGs with structure predictions (2718 out of 3922), structural assignments fully cover the sequence (with no uncovered sequence regions > 30 residues long). Within this set of fully covered SMOGs, the majority (2532 SMOGs, or 93%) includes a single covered region. Prevalence of SMOGs covered by a single structural domain shows a general correspondence between sequence-based modules and structural domains. The remaining 7% of fully covered SMOGs (186 SMOGs) include segments that can be split in multiple structural domains, pointing to inconsistencies between sequence-based domain decomposition and definition of structural domains in SCOP.

### Contradictions and errors in structure assignments

To assess potential errors and inconsistencies in DOGs, we analyze two types of contradictions between structure assignments to SMOG fragments and domain definitions by SCOP. First, we consider overlapping sequence regions in DOGs that are assigned different SCOP superfamilies. We find 2499 such cases, where two DOGs with different superfamily assignments intersect within the same COG sequences. This number comprises ~8% of total 29818 possible DOG intersections (based on the number of DOGs in each COG). The vast majority of these cases involve assignment of related SCOP superfamilies to the same region (i.e. the covered segments in any individual SMOG sequence do not differ by more than 30 residues.). We reduced this set by excluding SCOP folds that are known to contain homologous superfamilies, such as multiple Rossmann-type folds, TIM-barrels, beta propellers, etc. After the reduction, only 218 overlapping DOG pairs are left. Manual analysis of these cases suggests that a major part (~40%) of these pairs still correspond to homologous superfamilies, or to mutidomain superfamilies with individual domains homologous to other superfamilies. In the remaining set, which includes real contradictions between superfamily assignments, we find two main sources of errors, both occurring in multidomain sequences. First, excessive alignment extension by PSI-BLAST can lead to incorrect structure assignment for unrelated fragments adjacent to homologous domains. Second, a wrong superfamily assignment can be made for a domain inserted in another domain (e.g. a CBS domain inserted into a TIM-barrel).

An interesting additional source of contradiction is a local structural and sequence similarity of functionally important regions within globally different domains [45]. In thirteen COGs, same sequence regions are assigned both to canonical 4Fe-4S ferredoxins of alpha+beta fold and to all-alpha ferredoxins. Although these two types of ferredoxins are unlikely to be related, PSI-BLAST detects a significant sequence similarity of their functional motifs around the Fe-S cluster-binding sites. Structure comparison [45] reveals a local structural similarity of these sites, although they are surrounded by completely different structural scaffolds.

As another type of contradiction between our domain assignments and domain definitions in SCOP, we consider SCOP domains split into multiple DOGs. To detect these contradictions, we analyze sequence regions from different DOGs that are mapped to adjacent parts of the same SCOP domain. We find such regions in 514 pairs of DOGs, which comprise 1.7% of total 29818 DOG pairs sharing the same COG. Most of these DOG pairs belong to the same SMOG, suggesting that SMOG boundaries rarely cut a SCOP domain. There are only fifteen cases of adjacent DOGs from different SMOGs, with SCOP domain being split by the boundary between sequence-based ADDA modules.

Thus, the number of contradictions introduced by structure assignments to SMOG fragments is reasonably low. These contradictions are mainly due to the errors in automated domain delineation and sequence comparison, and to the inconsistencies between sequence-based comparison and SCOP classification. These inconsistencies are caused by homology between many SCOP superfamilies and by the presence of multidomain fragments in SCOP.

### Growth of total number of assigned SCOP superfamilies with number of solved SMOGs

**Figure 2:**
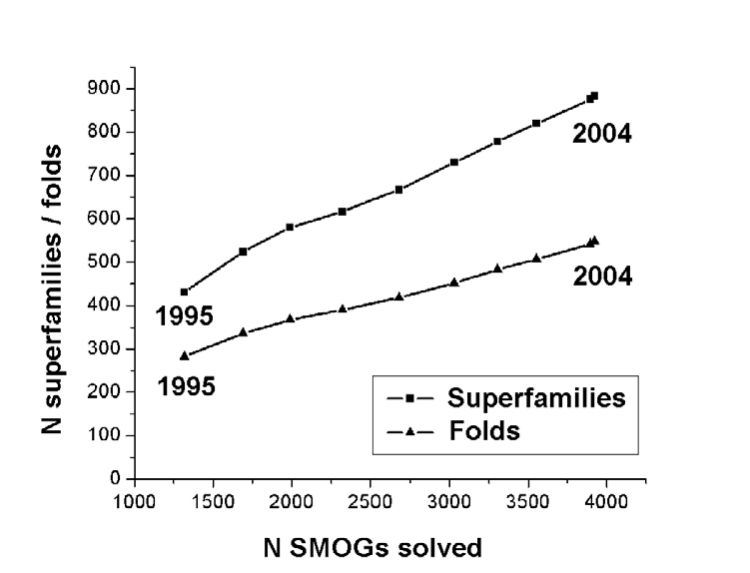
**Total number of assigned SCOP superfamilies and folds as functions of the number of solved SMOGs**. Each point represents a year, from 1995 to the present. See text for details.

Given today's structure assignments, how can one predict the total number of SCOP superfamilies and folds in the whole COG database? The simplest approach is to follow the growing number of assigned superfamilies with more SMOGs "solved" each year, and to extrapolate this growth to the total SMOG set. Figure 2 shows the numbers of different SCOP superfamilies and folds in solved SMOGs as functions of the number of solved SMOGs, each point representing a year from 1995 to 2004. (The recent version of SCOP 1.67 released in May 2005 includes only a small fraction of domain structures deposited in PDB in 2004; hence there is only a small difference between sets of SMOGs solved by years 2003 and 2004.)

These plots provide at least two observations. First, the number of solved SMOGs has tripled from 1995 till 2004, and comprises approximately a third of all SMOGs. Meanwhile, the total numbers of assigned SCOP superfamilies and folds in COG have doubled, comprising approximately 900 superfamilies and 550 folds. Second, both the numbers of newly solved SMOGs and of newly assigned superfamilies/folds stay approximately the same each year (250 to 300 SMOGs, ~50 superfamilies, and ~30 folds, respectively). This linear growth is in contrast with the exponential growth of the number of solved individual structures and emphasizes the high redundancy of currently solved structures in terms of homology-based structure prediction. Given the current numbers of newly solved SMOGs per year, all 13500 SMOGs would be solved in 30 to 40 years. Extrapolating the plots in Fig. 2 as lines to the total number of SMOGs produces the estimates of ~2500 SCOP superfamilies and ~1400 folds in the whole dataset.

These simplistic estimates would be reasonable if the current population of solved SMOGs represented an unbiased sample of the whole SMOG set. However, this is not the case: there has been a general tendency to solve structures of families with larger numbers of homologous proteins, resulting in under-representation of smaller, less connected groups that frequently correspond to separate new superfamilies and folds. This bias makes the estimate by linear growth a conservative lower estimate. To evaluate this bias, we map the set of solved SMOGs onto the network of sequence-based connections between all SMOGs, and compare the resulting subgraph to the whole network.

### SMOGs with many links to other SMOGs are more likely to be solved

We link SMOGs to each other with various linkage stringency. As a criterion for linking SMOGs 1 and 2, we use the portion *W *of sequences in SMOG 1 that, as PSI-BLAST queries, provide detection of sequences in SMOG 2 with E-value below *E *(Fig. 1C, see Methods).

**Figure 3:**
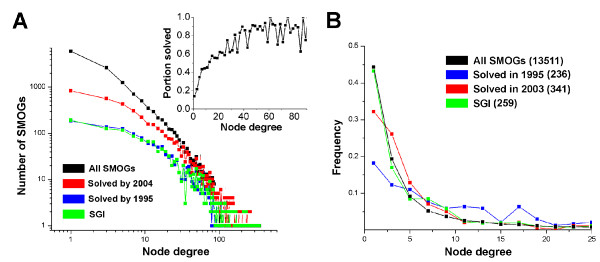
**Highly connected SMOGs are more likely to be solved**. Distributions of node degree for all SMOGs, compared to the population of solved SMOGs and to the SMOGs solved by structure genomic initiative. A. Distributions for cumulative SMOG populations solved by a certain  year (shown as absolute SMOG numbers on the log-log scale). SMOGs that are linked to the structures determined by year 1995 (blue), by year 2004 (red), and to the structures determined by structural genomics initiatives (green) are compared to the whole SMOG set (black). The inset shows the fraction of solved SMOGs for different bins of node degree. B. Distributions for SMOGs solved in a certain year and for SMOGs  initially solved by SGI (shown as frequencies). SMOGs that were for the first time linked to a structure solved in 1995 (blue), 2003 (red), and to a structure produced by SGI (green) are compared to the whole SMOG set (black). Number of SMOGs in each population is indicated in parentheses.

As nodes in the graph of sequence-based links between SMOGs, the sample of solved SMOGs is biased toward highly linked nodes. Figure 3A shows distributions of node degrees (numbers of links) for all SMOGs and for the sets of SMOGs solved in 1995 and in 2004 (distributions are shown as absolute numbers of SMOGs). Comparison of the 2004 graph to the total distribution shows that to date almost all SMOGs with >~30 links are solved, in contrast with ~20% among poorly linked SMOGs. A similar bias is observed at different stringencies of SMOG links, for all cutoffs of *E *and *W*. This bias probably reflects a greater interest of the structural community in solving proteins from larger families with many sequence-based homologs. Comparison of graphs in 1995 and 2004 shows that although with time this distribution becomes closer in shape to the total distribution, the bias toward highly connected SMOGs still persists. The set of all structures produced up to date by projects of structural genomic initiatives (SGI) also shows a similar bias in the population of covered SMOGs (Fig. 3A). However, the majority of these SMOGs was already linked to non-SGI structures solved earlier.

To assess how the bias toward highly linked SMOGs changes with time, we build the differential version of the node degree distribution. This distribution is based on the set of SMOGs that are solved exactly in a given year, i.e. SMOGs whose oldest assigned structures were deposited in PDB this year. Figure 3B shows the distributions of node degrees for SMOGs solved in 1995 and 2003, as compared to SMOGs that were first solved by SGI, and the total distribution for all SMOGs. The fraction of poorly linked SMOGs is higher in 2003 than in 1995, but the distribution is still skewed compared to the total. Interestingly, SMOGs that are first solved by SGI obey a distribution very similar to the overall set. This distribution is consistent with the random sampling of solved SMOGs from the whole population, reflecting a much smaller bias in the set of SGI targets compared to other solved structures.

### Relations between SCOP superfamilies and clusters of SMOGs

Given the over-representation of highly connected SMOGs in the solved population, we estimated the total number of SCOP superfamilies in the COG database by (i) clustering SMOGs by sequence similarity at various linkage stringencies, and (ii) monitoring and extrapolating into the future the relations between SMOG clusters and SCOP superfamilies.

SMOG clusters may roughly correspond to the superfamilies, but this correspondence is never perfect because (i) many SCOP superfamilies are related and sequence similarity between them can be detected; (ii) some superfamilies include distant homologs whose similarity can be detected only by structure analysis and not by PSI-BLAST; and (iii) unrelated superfamilies may be erroneously included in the same SMOG cluster by linking multidomain sequence segments or by spurious PSI-BLAST hits. Rather than improving the accuracy of domain prediction and similarity searches, we consider the last factor (errors of the automated methods) an inherent property of the network and assume that this factor is independent of time. We change the input from all three factors by varying the stringency of links between SMOGs, from no connections at all (separate SMOGs as clusters) to the most relaxed linkage criteria, and obtain estimates of total number of superfamilies for each stringency level.

We consider distributions of the number of superfamilies in a cluster (*f*_*m*_) and of the number of clusters covered by a superfamily (*g*_*n*_). These distributions allow for precise calculation of the total number of superfamilies *M *in a given set of solved SMOGs. The formula involves the total number of clusters *N *along with <*m*> and <*n*>, the average number of superfamilies assigned to a cluster and the average number of clusters corresponding to a superfamily (see formula (3) in Methods). To predict the total number of superfamilies M* in the whole SMOG set, we monitor the changes in distributions *f*_*m *_and *g*_*n *_with the growth of the solved SMOG set in time, make predictions <*m*>* and <*n*>* of <*m*> and <*n*> for the whole dataset, and apply formula (3), given the total number of SMOG clusters *N*. Since *N *is known exactly, and <*m*> and <*n*> change slowly, we expect the relative error of our estimate M* to be within a reasonable range (see Methods). To evaluate the consistency of our estimates, we consider SMOG clustering based on various linkage stringencies, make predictions of M* for each stringency, and compare the results. We apply the same considerations to the estimation of the total number of folds. However, since folds much more frequently include proteins with no sequence similarity, the relative error for the estimates of the number of folds should be larger than that for superfamilies.

### Distributions *f*_*m *_and *g*_*n*_

**Figure 4:**
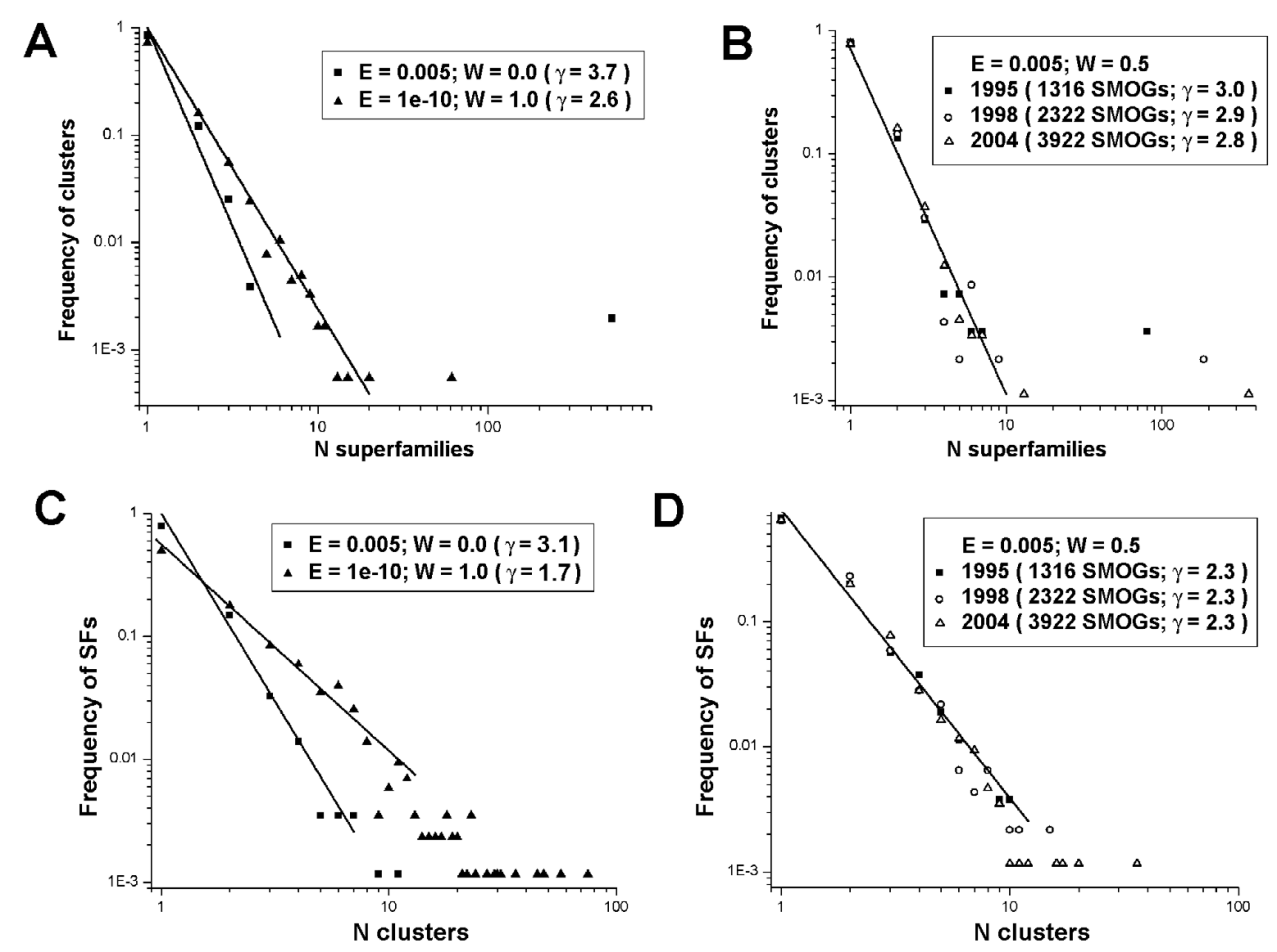
**Distributions of number of superfamilies in a SMOG cluster and of number of clusters with a given superfamily**. Distributions of number of superfamilies in a SMOG cluster (*f*_*m*_) shown as log-log plots, for various linkage stringencies (A) and for different sizes of the population of solved SMOGs over the years (B). Distributions of number of clusters with a given superfamily assigned (*g*_*n*_), for various linkage stringencies (C) and for different sizes of the population of solved SMOGs over the years (D). To illustrate the sharpness of the distributions, power-law approximations of the continuous parts are shown as lines, with their exponents (*γ*) indicated in graph legends. In B and D, the lines for different years are very close, and only a single approximation is shown, for the most recent population of solved SMOGs. See text for details.

Figure 4A shows distribution *f*_*m *_of the numbers of superfamilies *m *assigned to a SMOG cluster, for different linkage stringencies. The majority of SMOG clusters include a single superfamily. The distribution consists of two distinct parts: a rapidly decreasing part, which can be approximated by a power law *f*_*m *_~ *m*^-*γ *^(shown as a line on a log-log plot), and a single giant cluster. The continuous part is steeper than 1/*m*, so that *γ *> 1. Therefore, the contributions of various terms to the sum <*m*> = ∑mmfm
 MathType@MTEF@5@5@+=feaafiart1ev1aaatCvAUfKttLearuWrP9MDH5MBPbIqV92AaeXatLxBI9gBaebbnrfifHhDYfgasaacH8akY=wiFfYdH8Gipec8Eeeu0xXdbba9frFj0=OqFfea0dXdd9vqai=hGuQ8kuc9pgc9s8qqaq=dirpe0xb9q8qiLsFr0=vr0=vr0dc8meaabaqaciaacaGaaeqabaqabeGadaaakeaadaaeqbqaaiabd2gaTjabdAgaMnaaBaaaleaacqWGTbqBaeqaaaqaaiabd2gaTbqab0GaeyyeIuoaaaa@346E@ decrease with *m *as approximately *m*^1-*γ*^. Thus, the value of <*m*> is not significantly affected by possible aberrations in the tail caused by fluctuations in the small number of clusters with many assigned superfamilies.

The giant cluster includes superfamilies of highly populated folds (such as TIM barrels, Rossman-type folds), as well as non-related superfamilies added as parts of multidomain SMOGs or due to spurious PSI-BLAST hits. With more relaxed linkage criteria, the number of superfamilies in the largest component grows from being close to other smaller components (~60 superfamilies) for the stringent linkage to larger sizes (up to ~500) for more inclusive cutoffs (Fig. 4A). The growth occurs by the inclusion of other clusters in the largest component. This inclusion is more likely to happen to larger clusters, which makes the continuous part of the distribution steeper (Fig. 4A).

Figure 4B shows the changes of this distribution in time, for intermediate linkage stringency. There are two sources of these changes: inclusion of previously unsolved SMOG clusters and assignment of additional superfamilies to clusters with already solved SMOGs. With more structures solved, the slope of the continuous part of the distribution decreases, and the number of superfamilies in the giant cluster increases. However, because of the growing total number of solved clusters, the giant cluster represents a smaller fraction of clusters *f*_*m*_, which balances its contribution to the average <*m*> = ∑mmfm
 MathType@MTEF@5@5@+=feaafiart1ev1aaatCvAUfKttLearuWrP9MDH5MBPbIqV92AaeXatLxBI9gBaebbnrfifHhDYfgasaacH8akY=wiFfYdH8Gipec8Eeeu0xXdbba9frFj0=OqFfea0dXdd9vqai=hGuQ8kuc9pgc9s8qqaq=dirpe0xb9q8qiLsFr0=vr0=vr0dc8meaabaqaciaacaGaaeqabaqabeGadaaakeaadaaeqbqaaiabd2gaTjabdAgaMnaaBaaaleaacqWGTbqBaeqaaaqaaiabd2gaTbqab0GaeyyeIuoaaaa@346E@ and reduces the influence of the giant component on the growth of <*m*>.

Figure 4C shows distribution *g*_*n *_of the number of clusters *n *corresponding to a given superfamily, for different linkage stringencies. The majority of superfamilies correspond to a single cluster. The distribution decreases faster than 1/*m *(power law approximation *g*_*n *_~ *n*^-*γ *^is shown as a line on a log-log plot). Increasing stringency of linkage leads to the reduction of cluster sizes and results in more superfamilies being split between multiple clusters. Accordingly, the slope of the distribution decreases, and individual highly populated superfamilies (with up to ~80 clusters) appear in the tail (Fig. 4C).

Figure 4D shows the changes of distribution *g*_*n *_in time, for intermediate linkage stringency. These changes are much less pronounced compared to distribution *f*_*m *_(Fig. 4A). The slope of distribution *g*_*n *_decreases only slightly, while more individual highly populated superfamilies appear in the tail (Fig. 4D).

### Estimates of total numbers of superfamilies and folds

**Figure 5:**
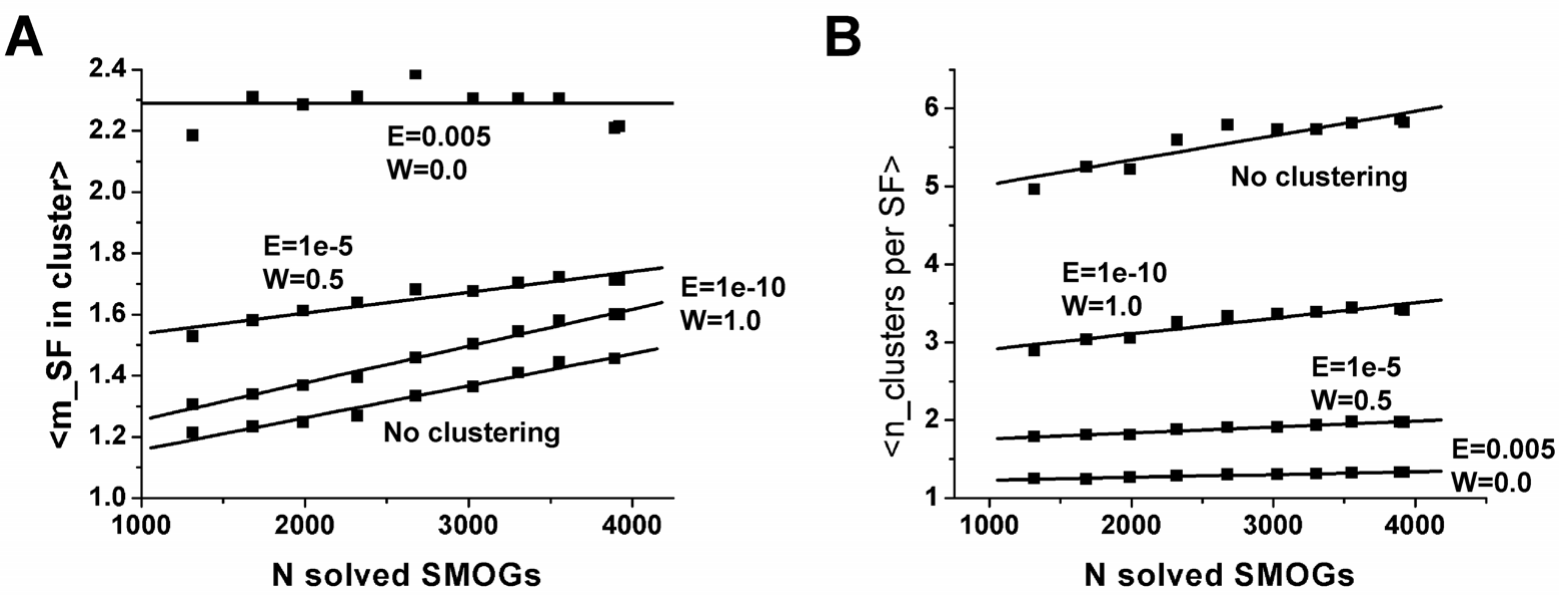
**Average numbers of superfamilies assigned to a SMOG cluster and of clusters corresponding to a superfamily**. Average number of superfamilies assigned to a SMOG cluster (A) and average number of clusters corresponding to a superfamily (B), for various linkage stringencies, plotted as functions of the number of solved SMOGs. Points in the graphs represent consecutive years, starting from 1995. Linkage stringencies are indicated as the cutoffs for E-value and W  (fraction of queries in SMOG 1 that provide PSI-BLAST detection of  sequences from SMOG 2). Graphs for separate SMOGs are marked as "No clustering".

The changes in distributions *f*_*m *_and *g*_*n *_(Fig. 4B,D) result in a relatively slow growth of both the average number of superfamilies assigned to a SMOG cluster <*m*> (Fig. 5A) and the average number of clusters corresponding to a given superfamily <*n*> (Fig. 5B). The values of <*m*> for the current population of solved SMOGs range from 1.4 for no links allowed (all SMOGs as separate clusters) to 2.3 for the most relaxed linkage cutoff (link formed between SMOGs with at least one PSI-BLAST hit below default E-value). The fastest growth of <*m*> is provided by the clusters with the most stringent linkage criteria: since 1995, the value has increased by 18%, which corresponds to ~2% per year and ~0.006% per solved SMOG. For more relaxed linkage criteria, this growth is slower (Fig. 5A).

The current values of <*n*> are between 1.3 for the most relaxed linkage and 5.7 for separate SMOGs (Fig. 5B). As in the case of <*m*>, the fastest growth of <*n*> is observed for the clusters with the most stringent linkage criteria: the maximal increase since 1995 is ~15%, which corresponds to <~2% per year and ~0.005% per solved SMOG. For more relaxed linkage criteria, this growth is slower; the slowest growth of ~0.7% per year (~0.2 10^-3^% per SMOG) is observed for the most inclusive linkage (Fig. 5B).

The shape of the observed curves and the slow relative rate of the growth allows for linear approximations. Extrapolating these curves to the total number of ~13500 SMOGs provides the estimates of <*m*>* and <*n*>* in the whole SMOG set (Table 2). Given these estimates and the total number of clusters for each linkage stringency, we calculate predicted total numbers of superfamilies. As shown in Table 2, all predictions fall in the range of 4150 ± 450 superfamilies.

In a similar fashion we make predictions of the total number of different folds. We consider the distributions of number of SCOP folds in a cluster and of number of clusters covered by a fold (not shown), and estimate their average values (<m_folds_> and <n>_fold_) for the moment when all SMOGs are solved (Table 2). These estimates show the same general correlation with the linkage stringency as <m>* and <n>* for superfamilies. Having <m_folds_> and <n>_fold _at various linkage stringencies, we calculate predicted total number of folds using formula (3). These predictions are in the range of 1700 ± 400 folds (Table 2).

## Discussion

Here, we analyze protein sequences from 66 complete genomes included in the COG database, make homology-based predictions of their structure, and monitor the dynamics of these predictions in time, with the accumulation of experimentally solved structure templates. These templates currently allow for structure prediction in approximately a third of sequence-based modules (SMOGs), a fraction approximately three times greater than it would have been possible to predict with the structure set available in 1995.

We find a significant bias in the sample of SMOGs with assigned structure, compared to the whole population of SMOGs. Targets chosen for structure determination tend to be located in highly populated areas of sequence space, where many homologous families can be found. In contrast, the overall set of SMOGs that were initially solved by structural genomic initiatives (SGI) is very similar to a random sample from the whole population. Although it contributes only to a minor fraction of all presently solved SMOGs, the growing SGI output in future should further reduce the sampling bias.

Since many SGI projects are aimed at determining structures of previously unknown folds, one might expect even the opposite bias toward "singleton" families among those solved by SGI. The apparent absence of such a bias in our data might possibly be attributed to several factors. First, the present-day size of this SMOG sample is still relatively small (259 SMOGs), and the opposite bias might become pronounced when the sample grows. Second, the set of unsolved SMOGs comprises the majority (~70%) of the overall population, and a targeted random selection of the unsolved SMOGs that are not connected to the solved families may produce a sample similar to the overall population. Third, some of individual SGI projects have different preferences in target selection, e.g. focus on a particular proteome, which may increase the representation of domains with many sequence homologs. Another possible systematic factor is the exclusion of the proteins experimentally challenging in terms of crystallization and structure determination, which might affect the distribution of solved SGI targets.

Many previously proposed estimates of the total number of different folds assume random sampling of families from a certain distribution of number of families per fold. Most of the suggested approaches are conceptually similar. Assuming a specific random model of sampling sequence families for structure determination, the observed distribution of families among folds is fitted with a certain analytical function. This function and its optimized parameters are considered to represent the population of folds and families in the whole protein world. The total number of sequence families is either estimated independently or based on the estimates of others. Finally, given this number and the proposed form of the distribution, the total number of folds is derived. Different assumptions about the random model, shape of the distribution, and total number of families resulted in varying estimates. Alexandrov and Go [15] assumed normal distribution (6700 folds), whereas Wang [23] assumed uniform distribution (400 folds; a later estimate by the same author under different assumptions was ~650 folds [24]). To reflect the currently observed prevalence of folds with one or few families, skewed model distributions were used: Zhang and DeLisi [26] assumed geometric distribution (~700 folds), Govindarajan *et al*. [20] used stretched exponent (~1500–2000 folds found in nature), Wolf *et al*. [25] used a logarithmic distribution (~1000 folds), whereas Coulson and Moult [19] modeled the distribution by a three-part function and provided varying estimates for different assumed numbers of families (2300 for 10000 families, 4500 for 23100 families, and 10000 for 50000 families, with the latter estimate proposed as the most relevant).

Here, given the observed bias in the sample of families with assigned structure, we choose a non-parametric approach to the estimation of the total numbers of different superfamilies and folds in the COG database. This approach has several main differences from those previously proposed. First, the sequences do not have to be assigned a single superfamily or fold. This consideration is more realistic, given the presence of related superfamilies and even folds in the current SCOP classification, and the possibility of multiple assignments to undetected multi-domain sequences. Second, our approach does not assume the solved families to be a representative random sample of the family population. Third, instead of a single grouping of protein sequences into families, we produce various groupings (SMOG clusters) that emerge at different clustering stringency. Finally, although our approach requires a rough correspondence between the sequence-based clustering and SCOP classification, we allow for errors in the inference of sequence similarity. These errors lead to the emergence and approximately linear growth of the giant cluster, consistent with a constant low probability for a certain SMOG to be included in the giant component. We consider such errors an inherent random noise and assume that their frequency stays approximately the same in time.

Our approach allows for a bias in the sample of families with solved structure and does not require the bias to be constant in time. However, our extrapolation assumes that the change of this bias is gradual, the assumption supported by the currently observed data (Fig. 3). This assumption provides for continuity in the changes of distributions *f*_*m *_and *g*_*n *_(Fig. 4B,D) and relatively slow changes of the average numbers of superfamilies and folds per sequence cluster (<*m*>) and of the average number of clusters with a superfamily or fold assigned (<*n*>, Fig. 5). Another important assumption is that clustering by sequence similarity is loosely similar to the grouping of domains in superfamilies/folds, which leads to steep continuous parts in distributions *f*_*m *_and *g*_*n *_declining faster than 1/*m*. This steep decline ensures that the average values <*m*> and <*n*> are hardly affected by potential aberrations in the tail, where individual "large" clusters and superfamilies/folds are located. Although the distributions become less sharp with more structures solved, as reflected by the decrease in the exponent *γ *of power-law approximation (Fig. 4B,D), this modest decrease is not likely to have a serious effect on the contribution of the tail in the future

We pay special attention to estimating the number of SCOP superfamilies because this level of classification presents a more tractable grouping of protein world. By definition [12], superfamilies include homologous domains, with homology inferred from sequence and structure comparison. Grouping into superfamilies has fewer deviations from purely sequence-based clustering than grouping into folds, which does not imply homology. This provides for less uncertainty in the estimate of the total number of superfamilies. A more general reason for our attention to the superfamily level is that the fold category, loosely defined as major types of structure topology, leaves much more space for subjective interpretation, and classification of proteins into folds is less based on the internal properties of the protein set, such as evolutionary connectivity in the case of superfamilies.

In this work, we build on the initial high-quality grouping of proteins from the whole genomes provided by the COG database. Confining the protein set to COG makes our consideration more tractable but puts more restrictions on the results. In particular, our results are valid for major widespread protein superfamilies and folds that are included in the COG database. The COG database [29] does not include smaller families with two or less orthologous representatives in different genomes, which amounts to ~25% of all individual sequences in the genomes considered. Since COG includes mainly prokaryotic genomes, our results may not cover exclusively eukaryotic superfamilies and folds. According to the whole-genome surveys [36,37], such folds comprise 15–18% of all SCOP folds. Thus, our estimates may serve as lower bounds of the total numbers of superfamilies and folds in the whole protein world. The distance between these bounds and the optimal estimates depends on how well the COG database represents the whole population of protein folds.

Although each newly sequenced genome adds a number of new proteins with no detectable sequence similarity to other proteins, the 3D structures of such "singletons" suggest that they usually possess already known structural folds (for instance, [38-41]). Thus, proteins in the COG database probably provide a representative sample of major structural folds, and the presented estimates may be fairly close to the total numbers of major protein folds and superfamilies.

## Conclusion

We present the estimates of the total number of structural superfamilies and folds in the COG database of protein sequences from 66 complete genomes. Mapping protein domains with predicted structure onto the graph of sequence-based connections between all domains, we found that the choice of targets for structure determination is biased towards more populated regions of sequence space. This bias is absent among the subset of targets whose structure was initially solved by structural genomics initiatives. The total number of structural superfamilies and folds in the COG database are estimated as ~4000 and ~1700, which is respectively four and three times higher than the numbers of superfamilies and folds that can currently be predicted in COG proteins.

## Methods

### Identification of sequence modules (SMOGs) in COG proteins

To identify independently recurring sequence modules in COG proteins, we used the ADDA database [31] produced by Automatic Domain Decomposition Algorithm [30]. ADDA is based on identification of high-scoring continuous segments in all-to-all pairwise alignments in the non-redundant sequences of the NCBI NR database.

To reduce the amount of computation, we filter each COG separately by sequence identity using cd-hit program [42], and choose representatives that are <50% identical to each other. This filtering results in 84133 representatives out of the total 144317 COG sequences. Proteins in the COG database were matched to sequence segments in ADDA database, and the resulting segments were identified as "sequence-based" domains. The sequence similarity between segments in the same COG that were assigned to the same family by ADDA was verified by PSI-BLAST (single iteration run of PSI-BLAST 2.2.6 with default parameters, except for database size (-z) set to that of the current NCBI NR database, using profiles produced from the sequence segments as queries. The profiles were produced by 5 iterations of PSI-BLAST search with default parameters against the NR database. To verify the similarity, we demand the default PSI-BLAST Evalue of 0.005 and >50% coverage in the shortest of two compared sequences). Similar segments within each COG were grouped together. Sequence regions more than 30 residues long that did not match ADDA database were clustered in separate groups within each COG by sequence similarity detected by PSI-BLAST, with the default E-value cutoff and a stringent coverage cutoff (75% in the shorter sequence). This initial grouping produced 18778 groups in 4873 COGs, which included 115287 sequence segments. We further linked tightly connected sequence groups from different COGs. To link two groups to each other, we demanded that all segments of group 1, used as PSI-BLAST queries, find segments of group 2, with default PSI-BLAST E-value and >50% coverage in the shortest of the two compared sequences. Based on these links, we merged groups from different COGs by complete linkage clustering, resulting in 13511 larger modules, which we call SMOGs (Sequence Modules from Orthologous Groups of proteins).

### Assignment of SCOP superfamilies to SMOG sequences

We used all individual SMOG segments as queries for BLAST, RPS-BLAST, and PSI-BLAST [32,33] searches against the domains included in SCOP 1.67 and represented in ASTRAL [34,35]. Although ADDA has been shown to be one the most accurate automated methods for sequence-based domain decomposition [30], we find that a considerable portion of SMOGs still contains multiple domains. A query sequence that includes more than one domain may produce spurious hits due to alignment extension over domain boundaries and possible erroneous inclusion of dissimilar domains that are adjacent to the truly similar domains. This problem is magnified in the iterative searches, which repeatedly use sequence profiles constructed from alignments produced in the previous iteration. To reduce the effect of multidomain sequences and profiles, we perform similarity searches in several steps. At the first step, we construct the database of SCOP domain sequences and the database of profiles based on ASTRAL representatives with less than 40% identity to each other, and with transmembrane, coiled coil, small, low resolution structures, peptides, and designed domains excluded. For the profile construction, we run 5 iterations of PSI-BLAST 2.2.6 with default parameters against the NCBI NR database. These SCOP-based sequence and profile sets contain a very low portion of multidomain entries, given the high accuracy of manual domain assignment in SCOP [43]. Using SMOG segments as queries, we perform BLAST and RPS-BLAST searches in these databases, and select statistically significant hits that cover more than 50% of SCOP domain length. (In all searches, the effective database size for E-value calculation (-z) is artificially set to that of NCBI NR database, ~4.37·10^8 ^letters, in order to use standard E-value cutoffs adjusted for this database.) We assign corresponding SCOP superfamilies to the regions of SMOG sequences that are included in these alignments. Based on PSI-BLAST alignments of sequences within the SMOG, we propagate the superfamily assignments to the sequences of the same SMOG that do not produce direct hits to domains of this superfamily. As a result, we obtain an initial set of sequence regions associated with SCOP superfamilies, which allows us to make the first approximate delineation of domain boundaries in multidomain SMOGs. At the second step, we split SMOG sequences along these boundaries and use the resulting fragments to build PSI-BLAST profiles from homologous sequences detected in the NCBI NR database (Fig. 1B). We use these profiles as queries for PSI-BLAST searches against (i) SCOP domains and (ii) all other such fragments in SMOGs. Based on the sequence similarities found in this searches, we (i) map SCOP domains on the regions in SMOG fragments and (ii) map different SMOG fragments on each other. Using mapping (ii), we propagate structure assignments to similar regions of other fragments within the same SMOG. Finally, in each SMOG we consider sequence regions that are assigned the same SCOP superfamily and cluster these regions by sequence similarity (criterion for linking two regions: PSI-BLAST E-value < 0.005, PSI-BLAST  alignment covering > 50% of the shorter region, the number of covered  residues being > 30). We call the resulting groups of sequence regions with the same superfamily assignment "Domains from Orthologous Groups of proteins" (DOGs). The table of sequence regions in DOGs and SCOP superfamily  assignments to these regions is available at   .

The listing of structural genomic targets in the PDB database, as of July 2005, was obtained from [44].

### Linking SMOGs by sequence similarity with varying stringency

We link SMOGs to each other based on similarity of their individual sequences detected by PSI-BLAST. As the criterion to form a link between SMOG 1 and SMOG 2, we use the portion *W *of sequences in SMOG 1 that, being used as PSI-BLAST queries, provide detection of sequences in SMOG 2 with E-value below a given level *E *(Fig. 1C). We vary the stringency of links by applying various cutoffs of these two parameters: *E *between 10^-10 ^and 0.005 (default cutoff in PSI-BLAST), and *W *between 0 and 1.0. For example, cutoffs of *E *= 0.005 and *W = *0 allow the formation of the link if PSI-BLAST detects similarity between any pair of sequences in the two SMOGs, whereas cutoffs of *E *= 10^-10 ^and *W *= 1.0 require that all sequences of SMOG 1 provide PSI-BLAST detection of sequences in SMOG 2 with very low E-values. For various cutoffs, we analyze unweighted undirected graphs comprised of SMOGs as nodes and their links as edges.

### Deriving total number of superfamilies/folds from the distributions of the number of superfamilies/folds in cluster and the number of clusters per superfamily/fold

To make estimates of the total number of SCOP superfamilies in the COG database, we consider single-linkage clusters of SMOGs at various linkage stringency (defined by parameters *E *and *W*), and their relation to SCOP superfamilies. We consider the distributions of number of superfamilies in a cluster and of number of clusters covered by a superfamily. These two distributions allow derivation of total number of superfamilies from total number of clusters in a given SMOG population. Indeed, the number of all superfamily assignments to all clusters can be written as

Ω=∑mmnm=N∑mmfm     (1)
 MathType@MTEF@5@5@+=feaafiart1ev1aaatCvAUfKttLearuWrP9MDH5MBPbIqV92AaeXatLxBI9gBaebbnrfifHhDYfgasaacH8akY=wiFfYdH8Gipec8Eeeu0xXdbba9frFj0=OqFfea0dXdd9vqai=hGuQ8kuc9pgc9s8qqaq=dirpe0xb9q8qiLsFr0=vr0=vr0dc8meaabaqaciaacaGaaeqabaqabeGadaaakeaacqqHPoWvcqGH9aqpdaaeqbqaaiabd2gaTjabd6gaUnaaBaaaleaacqWGTbqBaeqaaOGaeyypa0JaemOta40aaabuaeaacqWGTbqBcqWGMbGzdaWgaaWcbaGaemyBa0gabeaaaeaacqWGTbqBaeqaniabggHiLdaaleaacqWGTbqBaeqaniabggHiLdGccaWLjaGaaCzcamaabmaabaGaeGymaedacaGLOaGaayzkaaaaaa@44DB@

where *n*_*m *_is the number of clusters that have exactly *m *superfamilies assigned, *f*_*m *_is the frequency of such clusters in the whole cluster set, and *N *is the total number of clusters in the set. On the other hand,

Ω=∑nnmn=M∑nngn     (2)
 MathType@MTEF@5@5@+=feaafiart1ev1aaatCvAUfKttLearuWrP9MDH5MBPbIqV92AaeXatLxBI9gBaebbnrfifHhDYfgasaacH8akY=wiFfYdH8Gipec8Eeeu0xXdbba9frFj0=OqFfea0dXdd9vqai=hGuQ8kuc9pgc9s8qqaq=dirpe0xb9q8qiLsFr0=vr0=vr0dc8meaabaqaciaacaGaaeqabaqabeGadaaakeaacqqHPoWvcqGH9aqpdaaeqbqaaiabd6gaUjabd2gaTnaaBaaaleaacqWGUbGBaeqaaaqaaiabd6gaUbqab0GaeyyeIuoakiabg2da9iabd2eannaaqafabaGaemOBa4Maem4zaC2aaSbaaSqaaiabd6gaUbqabaaabaGaemOBa4gabeqdcqGHris5aOGaaCzcaiaaxMaadaqadaqaaiabikdaYaGaayjkaiaawMcaaaaa@44DC@

where *m*_*n *_is the number of superfamilies that are assigned to exactly *n *clusters, *g*_*n *_is the frequency of such superfamilies in the whole superfamily set, and *M *is the total number of superfamilies in the set. Comparing these two equations, *M *can be calculated as

M=N∑mmfm∑nngn=N<m><n>     (3)
 MathType@MTEF@5@5@+=feaafiart1ev1aaatCvAUfKttLearuWrP9MDH5MBPbIqV92AaeXatLxBI9gBaebbnrfifHhDYfgasaacH8akY=wiFfYdH8Gipec8Eeeu0xXdbba9frFj0=OqFfea0dXdd9vqai=hGuQ8kuc9pgc9s8qqaq=dirpe0xb9q8qiLsFr0=vr0=vr0dc8meaabaqaciaacaGaaeqabaqabeGadaaakeaacqWGnbqtcqGH9aqpcqWGobGtdaWcaaqaamaaqafabaGaemyBa0MaemOzay2aaSbaaSqaaiabd2gaTbqabaaabaGaemyBa0gabeqdcqGHris5aaGcbaWaaabuaeaacqWGUbGBcqWGNbWzdaWgaaWcbaGaemOBa4gabeaaaeaacqWGUbGBaeqaniabggHiLdaaaOGaeyypa0JaemOta40aaSaaaeaacqGH8aapcqWGTbqBcqGH+aGpaeaacqGH8aapcqWGUbGBcqGH+aGpaaGaaCzcaiaaxMaadaqadaqaaiabiodaZaGaayjkaiaawMcaaaaa@4C86@

where <*m*> and <*n*> are the average number of superfamilies assigned to a cluster and the average number of clusters corresponding to a superfamily. Note that both averages involve redundant sets, i.e. a superfamily that is assigned to several clusters enters <*m*> via each cluster, and a cluster with several assigned superfamilies enters <*n*> via each of these superfamilies.

To predict the total number of superfamilies M* in the whole SMOG set, we monitor the changes in distributions *f*_*m *_and *g*_*n *_with the growth of the solved SMOG set in time, make predictions <*m*>* and <*n*>* of <*m*> and <*n*> for the whole dataset, and apply formula (3), given the total number of SMOG clusters *N*. Since *N *is known exactly, formula (3) provides the relative error of estimate M*: ε_*M *_= ε_*m *_+ ε_*n*_, where ε_*m *_and ε_*n *_are the relative errors of extrapolated values <*m*>* and <*n*>*. Since the changes in the overall shape of the distributions *f*_*m *_and *g*_*n *_are slow, the changes of <*m*> and <*n*> are small compared to their absolute values. In fact, growth rates for <*m*> and <*n*> are lower than 2% a year (see Results, Fig. 5), an order of magnitude slower than a 10% growth rate for the direct count of superfamilies (Fig. 2). Therefore, the relative errors ε_*m *_and ε_*n*_, as well as ε_*M *_should be much smaller than the relative error of the extrapolated direct count. To evaluate the consistency of our estimates, we consider SMOG clustering based on various linkage stringencies, make predictions of M* for each stringency, and compare the results. The same considerations are valid for the estimation of the total number of folds, however with somewhat higher relative error.

## Abbreviations

COG, cluster of orthologous groups of proteins; SMOG, sequence module in orthologous group of proteins, DOG, domain from orthologous group of proteins; SGI, structural genomics initiatives.

## Authors' contributions

RIS carried out the theoretical considerations, computational experiments, analysis of the results and drafted the manuscript. NVG conceived of the study, and participated in its design and coordination. Both authors read and approved the final manuscript.

## Figures and Tables

**Table 1:** Statistics of structure predictions by taxonomic groups. For each group, the total number of sequences in COG (Total COG sequences) is shown, along with the number and fraction (%) of chosen representatives with <50% identity (Representatives), the number and fraction of these representatives with structure predictions (Representatives predicted), the number of SMOG sequence segments with predictions (SMOG segments predicted), the number and fraction of SMOG segments fully covered by regions of structure prediction (Fully covered), the number and fraction of SMOG segments covered by a single domain region, among fully covered (Single domain). The category of "Other Bacteria" includes the bacterial groups that are less represented in the COG database (*Deinococcus*-*Thermus*, *Thermotogae*, *Fusobacteria*, *Aquificae*, *Cyanobacteria*).

Group	Total COG sequences	Representatives (%)	Representatives predicted (%)	SMOG segments predicted	Fully covered (% of predicted)	Single domain (% of fully covered)
***Bacteria***						
*Proteobacteria alpha*	23383	12997 (56)	8676 (67)	16681	6671 (40)	6329 (95)
*Proteobacteria gamma*	33375	12733 (38)	7977 (63)	15011	6234 (42)	5846 (94)
*Other Proteobacteria*	10979	5959 (54)	4015 (68)	7613	3108 (41)	2913 (94)
*Firmicutes*	20921	13626 (65)	9314 (68)	17249	7092 (41)	6641 (94)
*Actinobacteria*	9390	4241 (45)	3059 (72)	5741	2408 (42)	2257 (94)
*Chlamydiae – Spirochaetes*	2829	2039 (72)	1468 (72)	3078	1119 (36)	1029 (92)
*OtherBacteria*	13870	10670 (77)	7485 (70)	14905	5995 (40)	5680 (95)
***Archaea***						
*Euryarchaeota*	21118	14893 (71)	9968 (67)	19625	7625 (39)	7235 (95)
*Crenarchaeota*	1254	1131 (90)	774 (68)	1428	549 (38)	518 (94)
***Eukaryota***						
*Fungi*	7198	5880 (71)	2778 (47)	6801	3801 (56)	3616 (95)
**Total**	144317	84169 (58)	55514 (66)	108132	44602 (41)	42064 (94)

**Table 2:** Estimates of total numbers of superfamilies and folds. For various cutoffs of linkage parameters (W and E), total number of SMOG clusters (N) is shown, with the estimates for the average numbers of superfamilies and folds in a cluster (<m_SF_> and <m_folds_>), the average numbers of clusters with a superfamily and a fold assigned (<n>_SF _and <n>_fold_), and total numbers of superfamilies and folds (M_SF _and M_folds_).

W	E	**N**	<m_SF_>	<n>_SF_	**M_SF_**	<m_folds_>	<n>_fold_	**M_folds_**
No clustering		**13511**	2.47	8.90	**3750**	1.93	16.9	**1540**
1.0	10^-10^	**7237**	2.77	5.40	**3710**	2.21	9.62	**1660**
1.0	10^-5^	**6134**	2.74	4.52	**3720**	2.24	7.90	**1740**
1.0	0.005	**5464**	2.70	3.94	**3740**	2.22	6.99	**1740**
0.5	10^-10^	**5845**	2.72	3.46	**4590**	2.22	6.46	**2010**
0.5	10^-5^	**4900**	2.39	2.72	**4310**	1.83	4.86	**1850**
0.5	0.005	**4297**	2.37	2.62	**3890**	1.90	4.93	**1660**
0.0	10^-10^	**4886**	1.90	2.12	**4380**	1.42	3.76	**1850**
0.0	10^-5^	**3999**	2.23	1.80	**4510**	1.36	3.24	**1680**
0.0	0.005	**3364**	2.30	1.67	**4510**	1.26	3.04	**1390**
